# Watchful surgery in asymptomatic mitral valve prolapse

**DOI:** 10.3389/fcvm.2023.1134828

**Published:** 2023-04-12

**Authors:** Myriam Carpenito, Martina Gelfusa, Simona Mega, Valeria Cammalleri, Giovanni Benfari, Domenico De Stefano, Gian Paolo Ussia, Christophe Tribouilloy, Maurice Enriquez-Sarano, Francesco Grigioni

**Affiliations:** ^1^ Research Unit of Cardiovascular Science, Università e Fondazione Policlinico Universitario Campus Bio-Medico, Roma, Italy; ^2^ Section of Cardiology, Department of Medicine, University of Verona, Verona, Italy; ^3^ Research Unit of Diagnostic Imaging and Interventional Radiology, Fondazione Policlinico Universitario Campus Bio-Medico, Roma, Italy; ^4^ Department of Cardiology, Amiens University Hospital, Amiens, France; ^5^ UR UPJV 7517, Jules Verne University of Picardie, Amiens, France; ^6^ Valve Science Center, Minneapolis Heart Institute Foundation, Minneapolis, MN, United States

**Keywords:** mitral valve prolapse, degenerative mitral regurgitation, mitral regurgitation, asymptomatic patient, mitral valve surgery

## Abstract

The most common organic etiology of mitral regurgitation is degenerative and consists of mitral valve prolapse (MVP). Volume overload because of mitral regurgitation is the most common complication of MVP. Advocating surgery before the consequences of volume overload become irreparable restores life expectancy, but carries a risk of mortality in patients who are often asymptomatic. On the other hand, the post-surgical outcome of symptomatic patients is dismal and life expectancy is impaired. In the present article, we aim to bridge the gap between these two therapeutic approaches, unifying the concepts of watchful waiting and early surgery in a “watchful surgery approach”.

## 1. Introduction

### 1.1. Putting the problem in perspective

Organic (primary) mitral regurgitation implies the presence of anatomic abnormalities affecting the leaflets or the sub-valvular apparatus. The most common etiology is degenerative (DMR) (1, 2), and consists of mitral valve prolapse (MVP). Less common etiologies include rheumatic heart disease, valve calcification because of aging, congenital diseases, and endocarditis (3).

**Figure 1:**
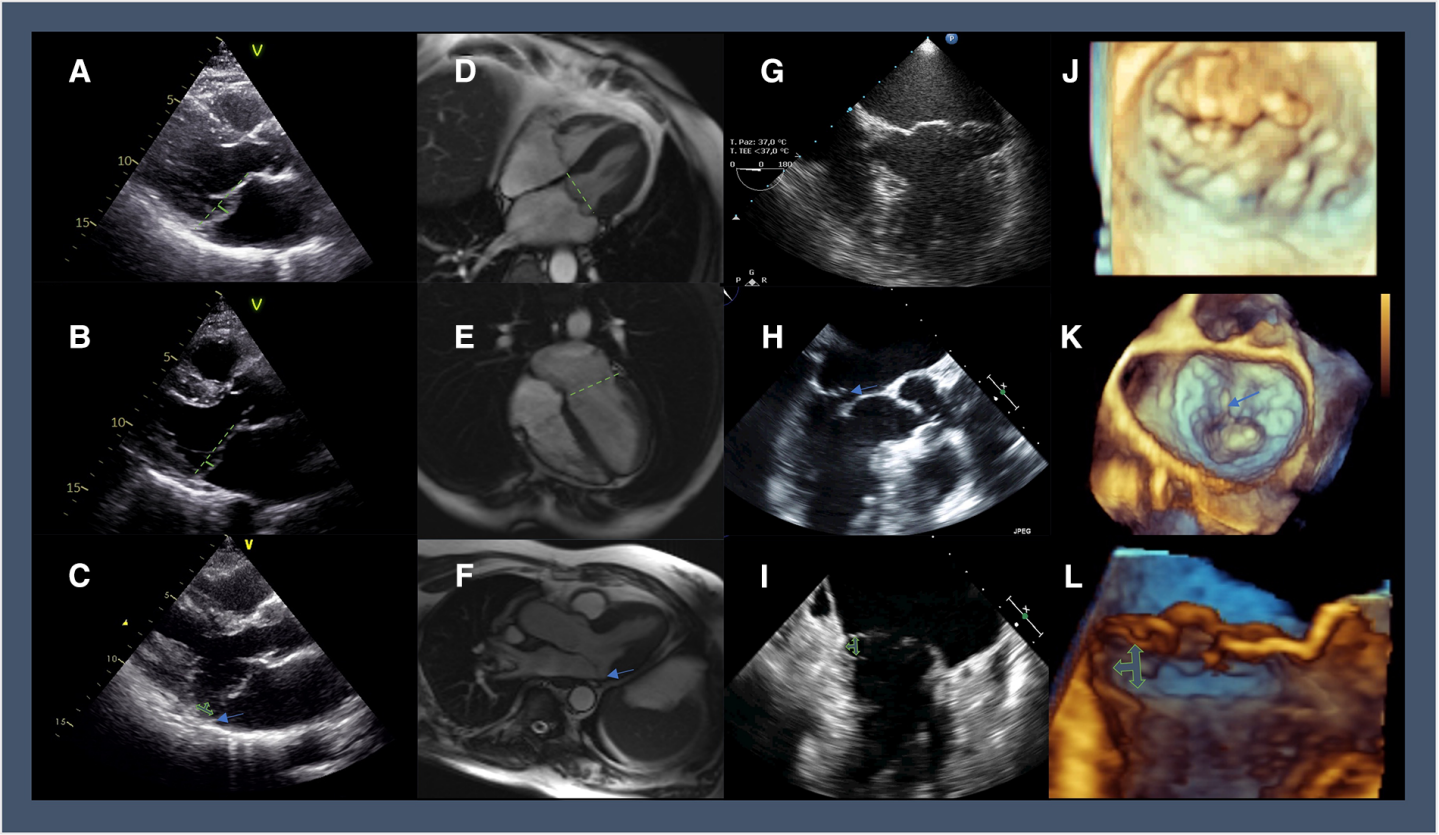
Multi-modality imaging highlighting the different features of MVP: 2D-TTE in parasternal long-axis view showing (**A**) diffuse thickening of the mitral tissue and the systolic displacement of posterior leaflets 3 mm beyond the plane of the annulus (green line); (**B**) leaflets thinning with a prolapse limited to posterior leaflets reaching cut-off of 2 mm (green line); (**C**) bileaflet prolapse and thickening of the mitral valve tissue with posterior MAD (green arrows; a blue arrow shows the detachment of postero-lateral annulus from posterolateral ventricular myocardium); Cine-CMR imaging in end-systolic 4-chamber view showing (**D**) Barlow's disease with bileaflet thickening of the mitral valve (green line representing the annulus plane); (**E**) fibroelastic deficiency with anterior leaflet thinning and prolapse in the left atrium; (**F**) MAD can be diagnosed in an SSFP three-chamber view in the systole (blue arrow); 2D-TEE (**G**) apical 4-chambers view showing Barlow's disease with a significantly enlarged mitral annulus and multisegmental prolapse and (**H**) 3-chamber of fibroelastic deficiency with P2 flail segment (blue arrow); (**I**) 2-chamber view of multiscallop prolapse with MAD (green arrow); 3D zoom aquisition of the mitral valve in surgical view showing Barlow's disease with multisegmental prolapse (**J**), and fibroelastic deficiency with P2 flail segment and ruptured chords (blue arrow) (**K**); cropped sagittal plane demonstrating a lateral view of the mitral valve with posterior MAD (green arrows) (**L**).

On the histological ground, infiltrative or dysplastic tissue disorders characterize MVP, with/without chordal rupture, producing two different phenotypes (4). At one hand of the spectrum, a significant excess of tissue, multi-scallop prolapse, annular dilation, and diffuse thickening/elongation of the mitral tissue indicate Barlow's disease. At the other end, leaflets thinning, chordal elongation, and prolapse limited to a portion of the leaflets set up the phenotype of fibroblastic deficiency (Figure 1).

On the physio-pathological ground, a systolic displacement of one or both mitral leaflets more than 2 mm beyond the plane of the annulus in a long-axis view defines MVP, with or without leaflet thickening. Two-dimensional transthoracic echocardiography (2D-TTE) is the most common diagnostic tool (Figure 1) (5). Based on these criteria, MVP prevalence in the general population ranges from 0.6% to 3.1% (6) and is higher in females (6, 7).

MVP may be syndromic, familial, or isolated (sporadic). We encounter syndromic MVP in connective tissue disorders such as Marfan syndrome, Loeys–Dietz syndrome, Ehlers–Danlos syndrome, pseudoxanthoma elasticum, osteogenesis imperfecta, and aneurysms-osteoarthritis syndrome (8). Among familiar patterns, autosomal dominant inheritance with varying degrees of penetrance is more common than the X-linked (9). Gender and age influence gene expression, with high phenotypic variability even within the same family (9). Isolated MVP is more frequently associated with benign extra-cardiac manifestations.

Since MVP is associated with such a composed group of underlying conditions and histological characteristics, its outcome is heterogeneous (10). Besides rhythm disturbances and endocarditis, volume overload is the most common complication, mostly corrected by conventional surgery. Surgery performed before the complications of volume overload restores life expectancy but implies a mortality risk in an asymptomatic patient. On the other hand, when surgery is performed in symptomatic patients with irreparable consequences of volume overload, the post-surgical outcome is dismal, and life expectancy is impaired.

In the present article, we will try to bridge the gap among these two (perhaps only apparently) diverging therapeutic approaches, trying to unify the opposite attitudes of “watchful waiting” (11) and “early surgery” (12) in a “watchful surgery approach”.

## 2. Before starting all thinking: is mitral regurgitation truly severe?

In MVP, the classic auscultatory finding is a dynamic mid-to-late systolic click, frequently associated with a high-pitched, late systolic murmur (13). A very loud murmur carries a high probability of severe DMR, but patients often present with a medium-intensity murmur, which can either be generated by moderate or severe regurgitation (14). Similarly, symptoms and cardiac remodeling support the diagnosis of severe DMR, but imaging is key if we want to offer surgery before the consequences of volume overload become irreparable. Doppler echocardiography, by providing qualitative, semi-quantitative, and quantitative parameters, represents the standard tool to diagnose the etiology, mechanism, and severity of any valvular lesion, including DMR (15). Grading DMR should be comprehensive, using a combination of clues, signs, and measurements (Table 1) (16). A detailed review of the different echocardiographic methods to establish the severity of DMR is behind the scope of the present article. However, we will summarize a few concepts applying to MVP.

**Table 1:** Echocardiographic features encountered in MVP and indicators of severe chronic regurgitation.

**Structural**	
LV remodeling	LVESD ≥ 40 mm
LA remodeling	Diameter ≥ 55 mm or volume ≥ 60 ml/m^2^
Leaflet thickening	Presence of flail leaflet
**Qualitative Doppler**	
Color flow jet area	Large central jet (>50% of LA) or eccentric wall-impinging jet of variable size
Flow convergence	Large throughout the entire systole
CWD jet	Dense signal holosistolic
**Semiquantitative**	
VCW (cm)	≥0.7 (>0.8 cm for biplane)
Pulmonary vein flow	Systolic flow reversal in more than one pulmonary vein
Mitral inflow	E-wave dominant (>1.2 m/s)
**Quantitative**	
ERO, 2D PISA (cmq)	≥0.4
RVol (ml)	≥60
RF (%)	≥50

CWD, continuous wave-doppler; ERO, effective regurgitant orifice area; LA, left atrial; LV, left ventricle; MVP, mitral valve prolapse; RF, regurgitant fraction; RVol, regurgitant volume; VCW, vena contracta width. Modified by Zoghbi et al. (16).

Systolic flow reversal in more than one pulmonary vein is specific for severe DMR, although eccentric jets can alter flow patterns, even mild or moderate in severity when directed into a pulmonary vein. The jet area or jet area/left atrial area ratio by color flow imaging is valuable as a screening tool to confirm the presence of more than mild DMR, but it is imprecise. This happens particularly in eccentric, wall-impinging jets and in late-systolic ones, as it can occur in MVP (16).

The vena contracta (VC) is the narrowest portion of the regurgitant flow and its measurement represents an approximation of the anatomic regurgitant orifice. A VC width ≥0.7 cm is specific for severe MR and this cut-off can be applied both to central and eccentric jets (16). As for color flow imaging, VC in MVP can overestimate the severity of late-systolic jets.

Concerning quantitative approaches, all methods derive three measures. The effective regurgitant orifice area (EROA) (a measure of lesion severity), the regurgitant volume per beat (RVol) (the severity of volume overload), and the regurgitant fraction (the ratio of the RVol to the forward stroke volume). Measurements of EROA and RVol provide the strongest prognostic information (17). In mid-late systolic jets, EROA by flow convergence appears similar to holosystolic. However, a shorter duration of regurgitation results in a smaller RVol. Herein, Rvol, rather than EROA provides more valuable information in this setting (18). When different parameters are contradictory, we must explain discrepancies. If uncertainties persist, transesophageal echocardiography or cardiac magnetic resonance (CMR) can help. On CMR, diffuse interstitial or regional replacement fibrosis by T1 mapping and/or late gadolinium enhancement and edema by T2 mapping can help identify the optimal timing of surgery in MVP complicated by severe DMR (19).

## 3. Is the patient truly asymptomatic?

Scientific guidelines recommend surgery (class I) in *symptomatic severe* DMR regardless of left ventricular (LV) function (15, 20) and the planned surgical procedure (e.g., repair/replacement). Before reviewing surgery in truly asymptomatic patients, it is worth noticing that several patients do not engage in a physical activity vigorous enough to reveal their symptoms.

To overcome this limitation, some studies analyzed the role of exercise as an additional prognostic indicator (21). Asymptomatic patients unable to exercise for at least 15 min on a treadmill using a modified Bruce protocol have a higher risk of adverse cardiac adverse events (22). Exercise echocardiography can provide additional information (20). A change in DMR severity (EROA ≥ 10 mm^2^, RVol ≥ 15 ml) by exercise echocardiography was associated with reduced symptom-free survival in more than moderate DMR (23). Similarly, exercise-induced right ventricular dysfunction (exercise TAPSE < 19 mm) and PASP > 55 mm Hg predict a worse outcome (24, 25).

A reduced peak oxygen consumption (<84% of expected) is common in DMR and is associated with adverse events (26). Although clinically appealing, the overall value of exercise-derived parameters in comprehensive DMR management remains to be properly tested. This is true particularly in the elderly, those with significant comorbidities, and—more in general- patients who cannot engage in intense physical activity.

## 4. Is there any consequence of volume overload?

### 4.1. Evaluating the left ventricle

The long-lasting physio-pathological dream in chronic DMR has been to identify the transition of the LV from the compensated phase (when LV is capable of managing the volume overload without permanent consequences if this is relieved by surgery) to the decompensated one (when the consequences of the volume overload are permanent) (27). In theory, the ability to diagnose this sweet spot would allow us to safely manage patients conservatively until the “real” need for surgery, without taking the risk of anticipating it when it is not necessary. Did this dream come through? Guidelines recommend (class I) surgery for DMR (regardless of symptoms and the planned surgical procedure e.g., repair or replacement) when the left LV ejection fraction (LVEF) is ≤60% or LV end-systolic diameter (LVESD) is ≥40 mm (15, 20). Although characterized by considerable interobserver variability of measurements (28), these two cut-offs should set the point in natural history when the risk of higher operative mortality and post-surgical ventricular dysfunction no longer justifies conservative management. Our group showed that patients with LVEF between 45% and 60% represent a large proportion of patients with DMR and, even if rarely symptomatic, they display a higher mortality rate and a worse post-surgical outcome as compared with LVEF > 60% (29).

LVESD is considered less loading dependent than LVEF, and a reliable indicator for surgery. Long-term multi-center studies in patients confirmed that LVESD > 40 mm (>22 mm/m^2^) represents a predictor of increased mortality under conservative management and after surgery (30). To further reduce the incidence of unexpected ventricular dysfunction after surgery, LVESD and LVEF have been combined, as a single indicator. Although this strategy is based on a valuable rationale, the occurrence of post-operative LV dysfunction remains notable (9% if LVEF ≥ 64% and LVESD < 37 mm, 21% if LVEF ≤ 64% or LVESD ≥ 37 mm, and 33% is LVEF ≥ 64% and LVESD ≥ 37 mm) (31).

Global longitudinal strain (GLS) of the LV is an early and sensitive method to detect dysfunction. In asymptomatic chronic DMR, the cutoffs to identify patients at high risk ranges from −17.9% to −21.7% (32). Further studies are needed to establish the clinical role of GLS in these patients. Similarly, three-dimensional echocardiography may represent a more reproducible and accurate method to assess ventricular size and function, but its incremental prognostic value over 2D-TTE in DMR needs confirmation (21).

Being ventricular function at rest modestly effective in predicting post-operative ventricular dysfunction, previous studies concentrated on exercise-derived parameters. In asymptomatic DMR, lack of contractile reserve (defined as an increase of 4% of LVEF) strongly predicts postoperative ventricular dysfunction (33). The absence of LV contractile reserve evaluated by GLS (exercise-induced increase less than 2%) may predict postoperative LV dysfunction (34).

### 4.2. Addressing the left atrium

In DMR, left atrial (LA) size is marginally affected by acute changes in preload and afterload, and its remodeling reflects the severity of volume/pressure overload over a longer period (35, 36). Our group showed LA size is a strong predictor of survival under non-surgical management and that LA volume ≥60 ml/m^2^ is a powerful prognostic indicator (37, 38). Accordingly, European guidelines indicate that a low-risk, effective, and durable mitral repair can be considered (Class II a) without any further risk factors when LA is ≥60 ml/m^2^ (diameter > 55 mm) (15, 39). LA function provides information (40). Among asymptomatic patients with preserved LVEF, both peaks of atrial longitudinal strain and reservoir strain may predict adverse outcomes (41). Left atrial coupling index (LACI) (represented by the ratio between LA volume index and tissue Doppler myocardial velocity during atrial contraction), emerged as a strong and independent determinant of the outcome under non-surgical treatment. LACI ≥ 5 was identified as a threshold for excess mortality (42). The novel finding that LA functional assessment is a meaningful marker of clinical outcomes underscores the need to standardize the atrial function assessment beyond morphology.

Besides being an indicator of overload severity, LA size negatively contributes to the outcome of DMR through the occurrence of atrial fibrillation (AFib). As the rhythm progress from sinus to paroxysmal to persistent AFib, the associated risk of mortality increases under non-surgical management, and the positive effects of surgery decrease in magnitude (43–45). Current European guidelines assign a Class IIa indication for surgery when Afib complicates DMR but a stronger strength of recommendation could be considered (15).

### 4.3. Challenging the pulmonary circulation

Pulmonary hypertension (PH) is a common complication of DMR and is related to increased LA pressure due to chronic volume overload. Abnormal pulmonary artery systolic pressure (PASP) independently affects prognosis, by causing right ventricular impairment (46), and subsequent functional tricuspid regurgitation (47). European guidelines recommend surgery when PASP at rest exceeds 50 mmHg (Class IIa) (15). A PASP value >50 mmHg is a powerful marker of poor prognosis in patients candidate for surgery (48). Recent data showed that an even milder increase in PASP (>35 mm Hg at rest) may predict early decompensation (49). In addition, the worsening of DMR during exercise and exercise-induced pulmonary hypertension is related to reduced symptom-free survival (23, 50). The presence of PH by Doppler echocardiography after correction of DMR is associated with late cardiac events and recurrence of symptoms (51). A more favorable postoperative outcome has been observed in patients who underwent surgery before the onset of PH (52, 53).

### 4.4. Circulating peptides

B-type natriuretic peptide (BNP) is released in response to increased myocardial wall stress (54, 55) and its amount of increase reflects DMR severity. Independent predictors of higher BNP plasma levels are LA and LV volumes, AFib, and PASP (56–61). A cut-off > 105 pg/ml of BNP may identify patients at higher risk (62). As BNP cut-off can vary significantly depending on patients' characteristics, a BNP ratio ≥1 (i.e., measured BNP to maximal expected normal value for age/gender and specific assay) is a powerful prognostic indicator in DMR independently of the other surgical triggers (63). BNP levels during exercise emerged as a marker of increased risk independently of baseline values and clinical/echocardiographic characteristics (64). When taken together, the pieces of evidence suggest that even if BNP is not yet included in guidelines, it may be considered in selected patients when the surgical triggers are contradictory and/or the timing of surgery uncertain.

## 5. Cardiac rhythm disturbances

Afib is not the only arrhythmic complication. MVP was identified as the cause of sudden cardiac death (SCD) in 4%–7% of young patients undergoing autopsy (65, 66). Arrhythmic mitral valve complex refers to MVP combined with frequent and/or complex LV arrhythmias in the absence of non-valvular pro-arrhythmic substrates (e.g., ventricular scar, channelopathy, etc..). The arrhythmic mitral valve complex includes patients *with and without severe D*MR (67). Risk factors for SCD in patients *with* MVP and severe MR are symptoms, Afib, and reduced LVEF (68). Mitral annular disjunction (MAD) is linked to LV arrhythmias in MVP regardless of DMR severity and is characterized by a systolic separation between the ventricular myocardium and the mitral annulus supporting the posterior leaflet (Figure 1) (69, 70). Depending on the diagnostic criteria, the prevalence of MAD in MVP varies between 20% and 58% (67). The origin of LV arrhythmias in MAD derives from the combination of the substrate (regional myocardial hypertrophy and fibrosis, Purkinje fibers) and the trigger (mechanical stretch) (66, 71, 72). In the first decade after diagnosis, MAD is not associated with increased mortality (73).

The stratification of SCD in MAD remains challenging and multiple indicators are currently under investigation. Late gadolinium enhancement localized on the LV infero-basal wall under the posterior leaflet, overlaps with myocardial fibrosis in the autoptic study in SCD victims (66). The prognostic significance of inducible arrhythmias in electrophysiological studies is currently unknown, and this test cannot be routinely recommended (71). While surgery is associated with a reduced risk of SCD in patients with severe DMR, its role in MAD without significant volume overload remains uncertain.

## 6. The watchful surgery approach

### 6.1. What is the quality of surgery I can offer?

No randomized prospective data are yet available but a large body of evidence collected at multiple centers worldwide consistently indicates that in DMR an *early, low-risk*, *effective* and *durable repair* is associated with lower in-hospital mortality, better survival, and lower long-term morbidity (12, 74–76). The advantages of minimally invasive surgery over conventional sternotomy –although relevant- are behind the scope of this paper. While European guidelines are more conservative in accepting medical management in asymptomatic patients without any risk factors (15), American guidelines recommend surgery providing a probability of a durable repair >95% and an expected operative mortality <1% (Class IIa) (20). A fundamental question is how often *all* these surgical requirements are satisfied in MVP. DMR includes patients with a limited alteration of the mid portion of the posterior leaflets as well as severe bi-leaflets prolapse. Such a heterogeneous anatomical spectrum conditions the difficulty of surgical procedures.

In terms of prevalence, an isolated posterior leaflet involvement is present in more than 80% of all comers receiving a diagnosis of severe DMR, complicating MVP (77). Consequently, a simple surgical correction (resulting in a higher likelihood of effective and durable repair) can be achieved in more than 90% of patients referred for surgery (78).

Concerning operative mortality, this was 1.7% overall in consecutive patients referred at tertiary centers for severe DMR, and lower after MV repair (1.3%) than after MV replacement (4.7%) (74). Operative mortality in asymptomatic patients with normal ventricular function undergoing mitral repair has been described in multi-center registries approaching 0% (79).

Concerning durability, the results for anterior and bi-leaflets prolapse are less favorable regarding the recurrence rate of moderate or severe regurgitation, with this risk approaching 1%–2% per year (80–84). Nevertheless, mitral repair after 20 years from the operation is overall characterized by better survival and a similar rate of re-operation as compared to replacement (less than 10%) (74).

Recently, transcatheter edge-to-edge repair emerged as an alternative to conventional surgical repair in high-risk patients unsuitable for surgery. Comparing patients treated with transcatheter and non-operated showed a significant advantage of percutaneous treatment with a prolonged higher survival rate (85).

### 6.2. Should I recommend surgery in truly asymptomatic patients with normal ventricular function and no consequences of volume overload without waiting any longer?

Yes, under few but stringent conditions.

The first condition is that DMR is truly severe. If uncertainties persist, we should take advantage of multiple diagnostic techniques or refer the patient to a heart valve center for a proper assessment.

The second condition is that an effective, low-risk, and durable repair can be achieved. If we cannot satisfy those requirements at our Institution, the referral of patients elsewhere represents the opportunity to improve and/or develop future internal dedicated surgical valve programs.

The third condition is that the procedure is not futile in light of a reduced life expectancy due to the presence of comorbidities. Percutaneous treatment is an option for these patients.

If multiple multi-center and single-center studies provided converging evidence that early surgical treatment is associated with a better long-term outcome (12, 76) data showing the overall safety and efficacy of a conservative approach taking into account post-surgical outcomes are lacking (11, 86).

## 7. Conclusions

DMR is a challenging yet tremendously exciting and evolving field of medicine. Thoughtful management of MVP demands a proper and comprehensive knowledge of genetics, hemodynamics, imaging, and arrhythmias substrates.

In the present article, we summarized old concepts, current orientations, and future perspectives applicable to everyday clinical practice.
